# Investigation of the Possible Protective Effect of *N*-Acetylcysteine (NAC) against Irinotecan (CPT-11)-Induced Toxicity in Rats

**DOI:** 10.3390/antiox11112219

**Published:** 2022-11-10

**Authors:** Sevgi Gençosman, Deniz Ceylanlı, Ahmet Özer Şehirli, Kerem Teralı, Furkan Bölükbaşı, Şule Çetinel, Serkan Sayıner

**Affiliations:** 1 Department of Biochemistry, Faculty of Veterinary Medicine, Near East University, 99138 Nicosia, North Cyprus, Turkey; 2 Department of Pharmacology, Faculty of Dentistry, Near East University, 99138 Nicosia, North Cyprus, Turkey; 3 Department of Medical Biochemistry, Faculty of Medicine, Cyprus International University, 99258 Nicosia, North Cyprus, Turkey; 4 Department of Histology and Embryology, School of Medicine, Marmara University, 34722 İstanbul, Turkey

**Keywords:** cancer chemotherapy, irinotecan, *N*-acetylcysteine, matrix metalloproteinase, proinflammatory cytokines, malondialdehyde

## Abstract

Irinotecan (CPT-11) is a chemotherapeutic agent involved in the treatment regimens for several malignancies such as colorectal cancer. *N*-acetylcysteine (NAC) is a strong antioxidant and anti-inflammatory agent used in the treatment of several diseases related to oxidative stress and inflammation. This study aimed at investigating whether NAC provides protection against hepatorenal and gastrointestinal tissue damage induced by CPT-11. Thirty-two Wistar albino rats were divided into four groups as control, NAC, CPT-11, and CPT-11+NAC. Following the experimental period, blood, and tissue samples (liver, kidney, stomach, and small intestine) were collected, and biochemical indicators, together with pro-inflammatory cytokines (TNF-α and IL-1β), matrix metalloproteinases (MMPs), malondialdehyde (MDA), glutathione peroxidase (GPx) and superoxide dismutase (SOD) levels were evaluated. Both the biochemical indicators and the pro-inflammatory cytokines, MMP, and MDA levels increased in animals treated with CPT-11, while SOD and GPx activities decreased. Histopathological evaluation revealed structural damage in all examined tissues. With NAC administration, significant improvements were observed, both biochemically and histologically. In conclusion, the results of the present study suggest that NAC treatment together with CPT-11 may have a beneficial effect on reducing CPT-11 toxicity in rats, by modulating inflammation and the oxidant–antioxidant balance. These results strongly promote further investigative studies.

## 1. Introduction

Globally, cancer is one of the leading causes of mortality. Chemotherapy (CT) and radiotherapy (RT) are currently the most effective and comprehensive approaches for cancer treatment [1]. However, they are known to have many adverse side effects, such as oral mucositis, gastrointestinal toxicity, hepatotoxicity, nephrotoxicity, hematopoietic system damage, cardiotoxicity, and neurotoxicity, and for these reasons their clinical use is limited [2,3,4]. These adverse effects generally reduce the quality of life and cause tapering of therapy in cancer patients. Therefore, it is highly critical to develop effective approaches and strategies against the side effects of CT and RT [5].

Irinotecan (CPT-11), is a semi-synthetic derivative of the plant alkaloid camptothecin (7-ethyl-10-[4-(piperdino)-1-piperidino]-carbonyloxycamptothecin) acting as a topoisomerase inhibitor. It is one of the most important anti-tumour drugs developed in recent years. While CPT-11 is used as a chemotherapeutic-cytotoxic agent with EMA (European Medicines Agency, EU)/FDA (Food and Drug Administration, US) approval in patients with colorectal and pancreatic cancer, positive effects have been seen in clinical studies in ovarian, cervical, lung and stomach cancers [6,7,8,9,10,11]. However, as with other chemotherapeutic agents, one of the treatment-limiting factors of CPT-11′s use is the increased chances of hepatorenal, and gastrointestinal damage. These common negative side effects of chemotherapy currently have no known cure or prevention [12]. In liver, kidney, stomach and intestinal tissues, treatment is limited, due to the increased levels of the lipid peroxidation product malondialdehyde, decreases in the level of antioxidant enzymes, increases in cytokine levels such as TNF-α and IL-1β, and the activation of matrix metalloproteinase enzymes, causing epithelial cell damage and congestion [13,14]. As a result, the emergence of anti-inflammatory drugs with antioxidant properties is helping to ameliorate these negative side effects.

*N*-acetylcysteine (NAC) is a mucolytic drug, first developed for acetaminophen (paracetamol) overdose, later gaining importance in many studies for its strong antioxidant effect. NAC is a small molecule with antioxidant capabilities that contains a thiol group [15]. NAC has been a widely used tool for investigating the redox sensitivity of biological or pathological processes [16]. It is known to increase the capacity to reduce pro-inflammatory cytokines and matrix metalloproteinases (MMPs). In previous studies, NAC’s antioxidant and anti-inflammatory effects have been shown to prevent tissue damage in non-alcoholic fatty liver disease [17], chronic kidney failure [18], and mobile phone radiation-induced oxidative stress [19].

Irinotecan is a widely used anti-chemotherapeutic agent and has many known side effects [20]. Various agents against irinotecan toxicity have been established in previous studies [21]. However, the need to test different agents in various study models, especially animal models, remains current. In the light of all these observations, it was aimed to examine the protective effects of NAC, which is a strong antioxidant, by measuring the effects of pro-inflammatory cytokine expressions, proteolytic enzymes, and antioxidant enzymes on the toxicity of irinotecan on liver, kidney, stomach, and small intestine toxicity.

## 2. Materials and Methods

### 2.1. Animals

The experimental protocol of this study was approved by the Local Animal Ethics Committee of Near East University (Protocol no. 127-2021/127). Thirty-two Wistar albino rats (250–300 g, male) were housed in a constant-temperature room (22 ± 1 °C) with a 12 h/12 h dark/light cycle. The rats had ad libitum access to food and drinking water.

The study animals were divided into four groups, with eight rats in each group (n = 8). Control group: the animals in this group were administered physiological saline solution (PSS) intraperitoneally (i.p.) on the first day and 0.01 mg/kg atropine subcutaneously (s.c.) (to prevent a possible cholinergic reaction to irinotecan) on the second day. CPT-11 group: PSS (i.p.) administered on day one to the animals in this group. On day two, atropine (0.01 mg/kg, s.c.) (to prevent a possible cholinergic reaction to irinotecan), followed by CPT-11 (125 mg/kg, i.p.) [22] administration. In the NAC group, animals were administered PSS (i.p.) on day one and atropine (0.01 mg/kg, s.c.) and a single dose of NAC (150 mg/kg, i.p.) [15]. CPT-11+NAC group: PSS (i.p.) administered on day one to the animals. On day two, atropine (0.01 mg/kg, s.c.) (to prevent a possible cholinergic reaction to irinotecan) followed by CPT-11 (125 mg/kg, i.p.) once [22], and a single dose of NAC (150 mg/kg, i.p.) [15], were administered.

### 2.2. Collection of Blood and Tissue Samples

Following the experimental period (48th hour after CPT-11 application), and following the loss of sensation after anaesthesia, the animals in all groups were euthanized by administering high-dose ketamine and xylazine (100 mg/kg and 10 mg/kg, respectively) [23]. Blood and tissue samples (liver, kidney, stomach, and intestine) were collected within 2–3 min for laboratory analysis. Blood samples were collected by cardiac vein puncture into vacuum tubes containing clot activators. Serum samples were obtained by centrifugation (K241, BRK5324, Centurion Scientific, Chichester, West Sussex, UK), at 1500× *g* for 10 min, after coagulation. Serum samples were stored at −80 °C until analysis. Tissue samples (stomach, small intestine, liver, and kidney) were collected for both biochemical and histopathological laboratory assays. After taking samples for biochemical analysis, erythrocytes were removed by perfusing with cold PSS. Then, the tissues were dried with filter paper and divided into two separate pieces for biochemical and histopathological analysis. Samples to be used for biochemical analysis were transferred to tissue sample cups with 50 mM phosphate buffer (pH 7.4) and transferred to the laboratory.

Tissue samples were transferred to the laboratory for further processes. Tissues were homogenized, following the manufacturer’s protocol using RIPA lysis buffer (item no. 10010263, batch no. 0490889-1, Cayman Chemicals, Ann Arbor, MI, US) and Dounce tissue grinder set (D8938, Lot. 3110, Sigma-Aldrich, St. Louis, MO, US) on ice. Then the homogenates were subjected to centrifugation at +4 °C at 10,000× *g* for 10 min (MIKRO 200R, Hettich, Tuttlingen, Germany), and the supernatants were portioned and stored at −80 °C, prior to analysis. Tissue sections to be used for histopathological examination were placed in containers containing 10% formaldehyde and kept at −80 °C until analysed [24,25,26]. The Bradford method was performed to quantify protein concentrations in tissue homogenates [27].

### 2.3. Measurements of Serum Enzyme Activities and Metabolites

Alanine transaminase (ALT), aspartate transaminase (AST), alkaline phosphatase (ALP), lactate dehydrogenase (LDH), lipase and amylase enzyme activities and total protein (TP), albumin, blood urea nitrogen (BUN), and creatinine tests were determined in blood samples, according to The International Federation of Clinical Chemistry IFCC protocols. Test parameters were measured using an automated chemistry analyser (BS-240 Vet, Mindray, Shenzhen, China).

### 2.4. Measurements of Pro-Inflammatory Cytokines

The concentrations of IL-1β and TNF-α in serum and tissue homogenates were determined by commercially available rat-specific ELISA assay kits (ELR-IL1β and ELR-TNF-α RayBiotech Inc., Norcross, GA, USA) following the manufacturer’s instructions.

### 2.5. Measurements of Matrix Metalloproteinases (MMPs) and TIMP-1

MMPs and TIMP-1, which play a role in many biological processes such as cancer and inflammation, were quantified in both tissue homogenates and serum, following the manufacturer’s instructions and the guidelines of the commercially available rat-specific ELISA assays (MMP-1 E-EL-R0617, Elabscience, Houston, TX, USA; MMP-2 ELR-MMP2; MMP-8 ELR-MMP8; TIMP-1 ELR-TIMP-1, RayBiotech Inc., Norcross, GA, USA).

### 2.6. Measurements of Antioxidant Enzymes and Lipid Peroxidation

GPx and SOD activities were measured using ready-to-use assay kits in tissue homogenates (stomach, small intestine, liver, and kidney) (RANSEL RS505, RANSOD SD125, Randox Laboratories Ltd., Crumlin, County Antrim, UK) by using an automated BS-240 VET Clinical Chemistry Analyzer (Mindray, Shenzhen, China).

MDA concentrations were measured in tissue homogenates (stomach, small intestine, liver, and kidney) to assess lipid peroxidation status, using commercially available assay kits (TBARS Assay Kit, item no. 10009055, Cayman Chemicals, Ann Arbor, MI, USA). The principal measurement was based on the reaction with thiobarbituric acid (TBA) in boiling water for 60 min in an acidic medium, and measurement of the absorbance of the reaction mixture at 532 nm [28]. Absorbance was measured with VersaMax Tunable Microplate Reader (Molecular Devices, San Jose, CA, USA).

### 2.7. Histopathological Evaluation

Tissues (stomach, small intestine, liver, and kidney) obtained immediately after the experimental period were fixed in 10% neutral formalin for 48 h, dehydrated in ascending alcohol series, and embedded in paraffin wax. Sections of 5 μm thickness were cut from the tissues, and routine hematoxylin and eosin staining was performed. Sections were examined histopathologically, using an Olympus BX50 photomicroscope (Olympus, Tokyo, Japan).

### 2.8. Statistical Analyses

Statistical analyses were conducted using the GraphPad Prism software (version 7.04, GraphPad Software, San Diego, CA, USA). All data were expressed as mean ± standard deviation (mean ± SD). An analysis of variance (ANOVA) was used to compare groups of data, and these were subsequently examined using Tukey’s multiple comparison tests. A difference of *p* < 0.05 was considered statistically significant.

## 3. Results

### 3.1. Serum Enzyme Activities and Metabolite Concentrations Restored by NAC

Serum enzyme activities (ALT, AST, ALP, LDH, amylase, lipase in U/L) and metabolite concentrations (BUN and creatinine in mg/dL; albumin and TP in g/dL) are shown in Table 1. ALT, AST, ALP and LDH activities, BUN and creatinine levels significantly increased in the CPT-11 group, compared with the control group (*p* < 0.001–0.0001). ALT, AST, ALP, and LDH activities, BUN and creatinine levels were significantly different between the CPT-11+NAC group and the CPT-11 group (*p* < 0.05–0.0001).

### 3.2. Activities of Tissue Antioxidant Enzymes and Concentration of Lipid Peroxidation Were Reduced by NAC

**Figure 1:**
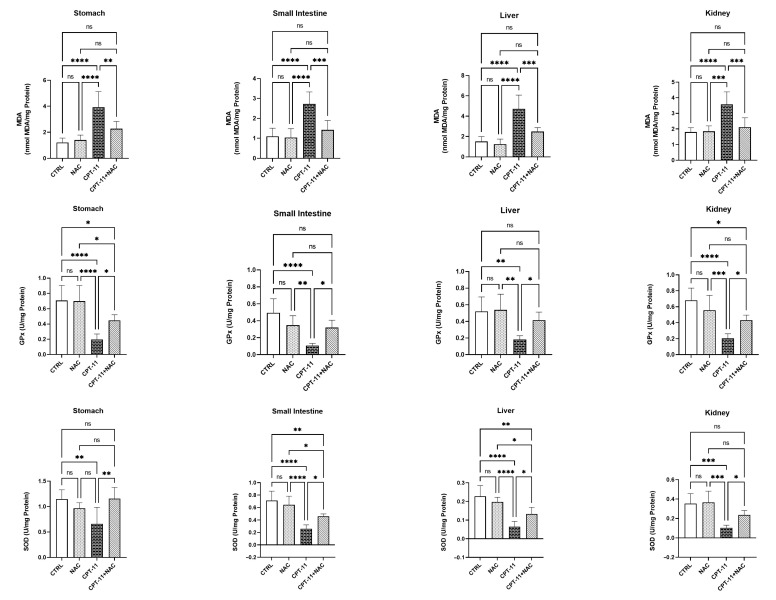
GPx, SOD activities and MDA concentration of stomach, small intestine, liver, and kidney tissue in experimental groups. * *p* < 0.05, ** *p* < 0.01, *** *p* < 0.001 **** *p* < 0.0001, ns: non-significant.

The activities of GPx, SOD, and the concentration of MDA in tissue homogenates (stomach, small intestine, liver, and kidney) are represented as U/mg protein (GPx and SOD) and nmol MDA/mg protein (MDA) in Figure 1. In comparison with both control and NAC groups, administration of CPT-11 significantly reduced GPx and SOD activities in the CPT-11 groups, in all tissue homogenates (*p* < 0.05–0.001). In addition, the GPx (small intestine, liver, kidney) and SOD (stomach, kidney) activities of the CPT-11+NAC groups were elevated to the levels of the control and NAC groups, and were significantly different from the CPT-11 group (*p* < 0.05–0.001).

However, the activities of GPx (stomach, kidney) and SOD (small intestine, liver) in the CPT-11+NAC group were increased, compared with the CPT-11 group (*p* < 0.05–0.01), although there remained a major difference when compared with the control group (*p* < 0.05–0.01).

In all tissue homogenates, MDA concentrations were remarkably elevated in the CPT-11 groups, compared with the control and NAC groups (*p* < 0.001–0.0001). Furthermore, there was a noticeable difference when compared with the CPT-11 group, in the MDA concentration of the CPT-11+NAC groups (*p* < 0.01–0.001) and in the increased concentration levels of the control and NAC groups in all tissues.

### 3.3. NAC Administration Decreased IL-1β and TNF-α Levels

**Figure 2:**
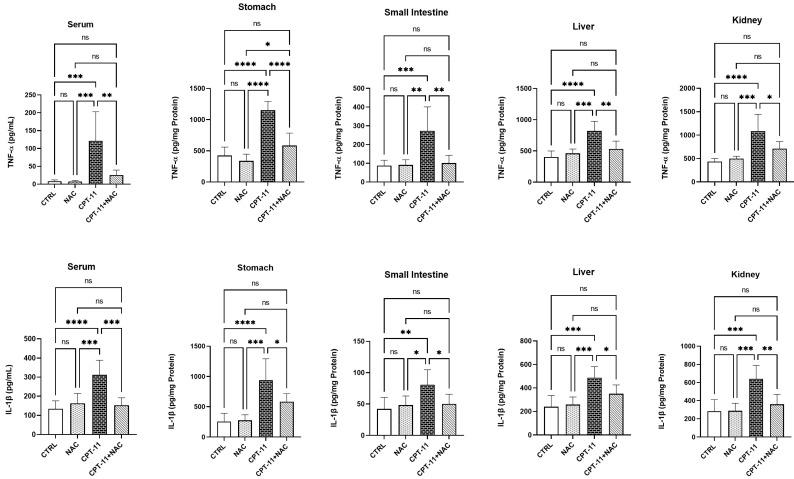
Serum, stomach, small intestine, liver and kidney levels of IL-1β and TNF-α in experimental groups. * *p* < 0.05, ** *p* < 0.01, *** *p* < 0.001 **** *p* < 0.0001, ns: non-significant.

Serum and tissue-homogenate (stomach, small intestine, liver, and kidney) levels of IL-1β and TNF-α are shown as pg/mL in serum and pg/mg Protein in tissue homogenates in Figure 2. In CPT-11 groups, CPT-11 administration significantly increased both serum and all tissue-homogenate levels of IL-1β and TNF-α, compared with both control and NAC (*p* < 0.05–0.0001) groups. IL-1β and TNF-α levels in the serum and all tissue homogenates reduced significantly in the CPT-11+NAC group, in comparison with the CPT-11 group (*p* < 0.05–0.001). However, no significant difference was observed between the experimental groups of control and NAC in the serum and all tissue homogenates.

### 3.4. NAC Reverses MMPs Activities and TIMP-1 Levels

The serum and tissue-homogenate activities of MMPs and levels of TIMP-1 are shown as pg/mL and pg/mg protein, respectively, in Table 2. CPT-11 administration significantly increased MMP-1, MMP-2, MMP-8 activities, and decreased TIMP-1 levels in the CPT-11 groups in serum and all tissue homogenates, compared with both control and NAC groups (*p* < 0.05–0.0001). Although there was a considerable difference in comparison with the CPT-11 group, the MMP-1, MMP-2, and MMP-8 activities of the CPT-11+NAC groups decreased, and the TIMP-1 levels of the CPT-11+NAC groups increased to the level of the control and NAC groups in the serum and all tissue homogenates (*p* < 0.05–0.0001).

### 3.5. Histopathological Findings

**Figure 3:**
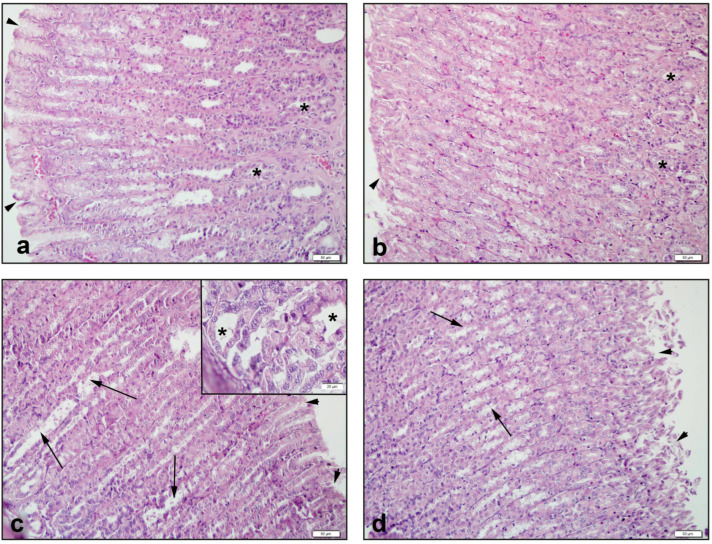
Histological view of stomach: (**a**) Control group of *: regular gastric glands and ‣: regular gastric epithelium, (**b**) NAC group of *: regular gastric glands and ‣: regular gastric epithelium, (**c**) CPT-11 group of *: irregular and enlarged gastric glands and ‣: gastric epithelium and →: openings between gastric epithelial columns, (**d**) CPT-11+NAC group of ‣: regression of openings between gastric epithelial columns and →: gastric epithelium. Hematoxylin & Eosin staining, 50 µm scale bar.

In the control group of the stomach, gastric tissue observations revealed a regular epithelial layer and gastric glands (Figure 3a). These structures were similar in the NAC group (Figure 3b). In the CPT-11 group, an irregular gastric epithelium and remarkable expansion of the gland structures were observed (Figure 3c). In the CPT11+NAC group, regression of openings of the gastric epithelial columns and improvement in gland structure were found (Figure 3d).

The stomach degeneration of the surface epithelium, disruptions in gland structures, and congestion scores increased considerably in the CPT-11 group when compared with the control and NAC groups (*p* < 0.01–0.0001). After treatment with NAC, the elevated stomach degeneration of the surface epithelium, disruptions in gland structures, and congestion scores, were significantly depressed (*p* < 0.05–0.0001) (Table 3).

**Figure 4:**
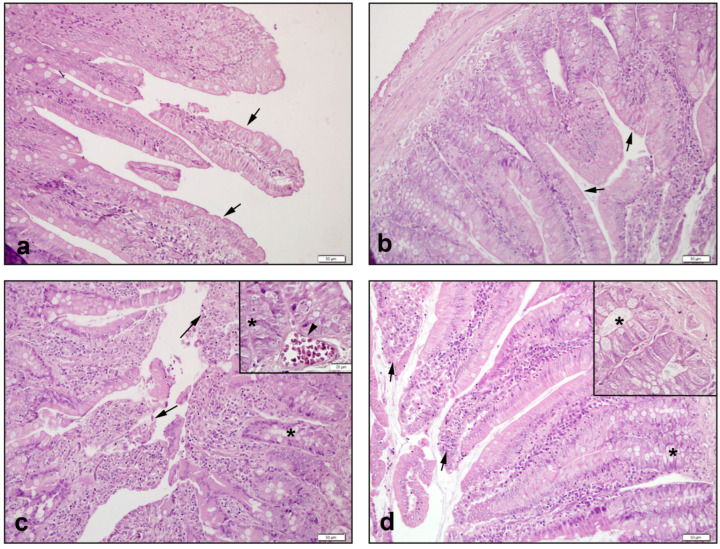
Histological view of small intestine: (**a**) Control group of →: villi structures observed with smooth intestinal epithelial cells; (**b**) NAC group of →: villi with smooth epithelial cells similar to the control group; (**c**) CPT-11 group of *: disruptions in the intestinal glands, and ‣: capillary congestion, and →: severe shedding of epithelial cells in villus structures; (**d**) CPT-11+NAC group of *: regression of disorders in the intestinal glands, and →: healings in villus epithelial cells. Hematoxylin & Eosin staining, 50 µm scale bar.

The control intestinal group (Figure 4a) and NAC group (Figure 4b) exhibited regular epithelial cells with regular villi and intestinal gland structures. In the CPT-11 group, severe shedding of villous epithelial cells, disruptions in the intestinal gland epithelium, and occasional capillary intravascular congestion were observed (Figure 4c). In the CPT11+NAC group, it was observed that the shedding of the villus epithelial cells regressed, and the intestinal gland epithelium healed (Figure 4d).

The small intestine villus degeneration, congestion in the lamina propria, and disruption of the intestinal glands score in the CPT-11 group, were found to be notably higher than the control and NAC groups (*p* < 0.01–0.0001). When NAC was administered, the elevations in small intestine villus degeneration, congestion in the lamina propria, and disruption of the intestinal glands scores were significantly suppressed (*p* < 0.05–0.001) (Table 3).

**Figure 5:**
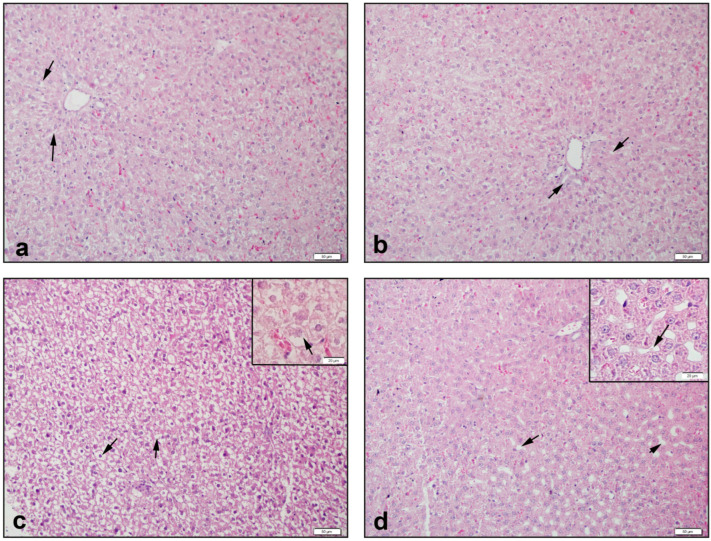
Histological view of liver: (**a**) Control group of →: regular hepatocytes and central vein in the middle; (**b**) NAC group of →: central vein and sinusoids; (**c**) CPT-11 group of →: hepatocytes that are severely enlarged and have lost their cytoplasm; (**d**) CPT-11+NAC group of ‣: hepatocytes were observed to regenerate, and →: the sinusoids returned to their former structures. Hematoxylin & Eosin staining, 50 µm scale bar.

Liver tissue was observed with smooth sinusoids and hepatocytes in the control group (Figure 5a). The NAC group also recorded images that were similar (Figure 5b). Severe enlargement of hepatocytes and loss of cytoplasm were observed in the CPT-11 group (Figure 5c). In the CPT-11+NAC group, it was observed that the loss of hepatocyte cytoplasm decreased, and sinusoids appeared (Figure 5d).

**Figure 6:**
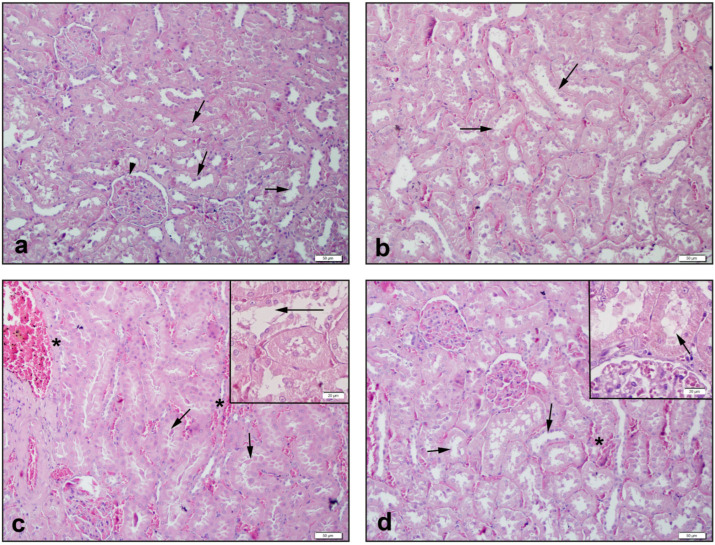
Histological view of kidney: (**a**) Control group of →: regular tubular and ‣: glomerular structures; (**b**) NAC group of →: regular tubular structures; (**c**) CPT-11 group of *: capillary congestion and →: severe shedding and caste formations in tubular cells; (**d**) CPT-11+NAC group of *: regression in congestion, and →: decreased shedding of tubule cells. Hematoxylin & Eosin staining, 50 µm scale bar.

Control kidney tissue had smooth glomerular and tubular structures (Figure 6a) and tissues in the NAC group showed similar images (Figure 6b). In the CPT-11 group, in addition to severe congestion and shedding of tubule cells, caste formations were also observed (Figure 6c). While regeneration of tubular structures was observed in the CPT-11+NAC group, it was observed that intratubular caste formations continued where the congestion regressed (Figure 6d).

In the CPT-11 group, degeneration in hepatocytes, sinusoids, and tubular cells, and kidney congestion scores showed a drastic increase (*p* < 0.05–0.0001), compared with the control and NAC groups. Conversely, the increase in scores significantly recovered in the CPT-11+NAC groups (*p* < 0.05–0.001) (Table 3).

## 4. Discussion

Today, despite all kinds of developments in the health sector, cancer cases are increasing, and the mortality rate remains high. Although significant progress has been made in cancer treatment through the application of chemotherapy, which began in the mid 20th century, the side effects of these chemotherapeutic agents are an important limiting factor in their use [29,30,31]. Therefore, both experimental and clinical studies on additional new drugs for the cancer treatment regimen, to reduce the negative effects of chemotherapeutic agents, are increasing. The chemotherapeutic agent CPT-11, a semisynthetic analogue of the natural alkaloid camptothecin, proved to be effective in patients with colorectal and pancreatic cancers, albeit with a high incidence of severe adverse effects. Hence, the purpose of this study was to investigate the possible ameliorating effect of NAC, a well-established antioxidant, on CPT-11-induced systemic toxicity in rats.

CPT-11 is metabolized by carboxylesterases (CESs) to form the pharmacologically active metabolites that bind and stabilize the topoisomerase I–DNA complex [32]. These metabolites are generally understood to be the main factors underlying the unique hepatorenal and GI toxicity of CPT-11 [17]. As is the case with other chemotherapeutic agents, CPT-11 has adverse effects. Hepatorenal and gastrointestinal symptoms are the most common issues caused by its use [33]. Currently, the major problem with CPT-11 use is that it causes hepatorenal injury and GI mucositis in patients; this is the most important factor limiting the dose of medication given to patients [34].

In the present study, it was observed that CPT-11 application changed AST, ALT, ALP and LDH activities, BUN, and creatinine levels in rat sera. It has been reported in the literature that increases in these parameters are evidence of changes at tissue and organ level [35,36]. Increases in AST, ALT, BUN, creatinine, LDH and ALP are markers of cellular damage in systemic tissues. Consistent with hepatorenal and gastrointestinal damage caused by CPT-11 application, a significant increase in serum enzyme activities was observed [36,37]. The serum activities of these markers were found to be significantly raised in the CPT-11 group, and NAC significantly reduced these increases and reversed CPT-11 toxicity. High activities of AST, ALT, LDH and ALP and high levels of BUN and creatinine in the blood may be an indicator of long-term systemic changes. Levels of these markers have been associated with hepatorenal and gastrointestinal tract damage [38]. Indeed, the histopathological findings obtained in this study support this observation. Other studies obtained similar histopathological findings, using different agents against the hepatorenal and gastrointestinal toxic effect of CPT-11 [39]. The CPT-11 application was determined to cause structural damage in stomach, small intestine, liver and kidney tissues, and this damage regressed following NAC treatment.

*N*-Acetylcysteine is a glutathione inducer and an antioxidant used for mucolytic purposes [18]. In addition to its mucolytic effects, it is used clinically, to scavenge the toxic metabolites that occur with the depletion of glutathione stores during paracetamol poisoning [40]. Many studies have shown NAC to have anti-inflammatory properties along with antioxidant properties, and for this purpose, it has been studied in various toxicity and inflammation models [17,18]. Although NAC reduces the toxic effects of chemotherapeutic agents, there are controversial findings suggesting it can reduce the effect of chemotherapeutic agents in cancer patients because reactive oxygen species (ROS) are required for the destruction of tumour cells. It is ironic that reactive oxygen species both cause and suppress cancer, as a result of chemotherapy. For example, in breast cancer cases, the survival of tumour cells depends on high ROS levels. Therefore, the reduction of these ROS levels is also important for the destruction of cancer cells. With these effects, NAC can show beneficial effects not only by looking at the negative effects of chemotherapy, but also by reducing the level of ROS needed by the cancer cell [41]. In this study, the main aim was to explore the protective effect of NAC against the toxicity caused by CPT-11, which has been used in cancer treatment in recent years. It was determined that inflammatory, lipid peroxidation, antioxidant and proteolytic enzyme markers examined with CPT-11 application were significantly negatively affected, whereas NAC application had a regenerative effect. In addition, the histopathological examination of stomach, small intestine, liver, and kidney tissues found results to be compatible with biochemical results.

TNF-α and IL-1β are cytokines with pleiotropic effects [42]. While they have a protective impact on the body with their effects on pathogens, their excessive secretion causes an inflammatory response and leads to tissue damage [43]. Because of these properties, they are known to be the main regulatory molecules in the physiopathology of inflammation. Additionally, it has been shown that the expression of these cytokines is associated with the disruption of oxidant–antioxidant balance, and increases the activities of proteolytic enzymes such as MMPs [44]. It has been suggested that NAC reduces pro-inflammatory cytokine expression in different experimental models [17,45]. In the current study, consistent with previous studies, it was observed that TNF-α and IL-1β levels increased in serum, liver, kidney, stomach, and intestinal tissues of rats, following CPT-11 treatment. However, NAC administration decreased cytokine expression in these tissues, thus providing supporting evidence for its anti-inflammatory effect.

MMPs are a family of proteolytic enzymes, and essential components of the extracellular matrix, with important roles in many processes, including tissue repair and inflammatory response [46]. Tissue inhibitors (TIMPs) are effective regulators of MMPs’ activities; in other words, they are natural MMP inhibitors [47]. They exert their inhibitory effects by non-specifically binding to MMPs [48]. Therefore, changes in MMP and TIMP levels determine the effectiveness of MMPs in contributing to tissue homeostasis [49]. Significant changes were detected in the levels of MMP-1, -2, -8 and TIMP-1, both in the blood serum as a reflection of the systemic response and in the hepatorenal and gastrointestinal tract tissues of animals treated with CPT-11. The relationship between MMP and TIMP levels in cases of inflammatory and oxidant–antioxidant imbalance has been shown in different study models [50]. On the other hand, it was found that these levels were reversed in the NAC-treated groups. Several models of inflammation have been used to study how NAC affects the activities of MMPs and the levels of TIMP-1 expression [51,52]. This suggests that CPT-11-derived MMPs are up-regulated and contribute to the damage, but NAC triggers down-regulation pathways with its antioxidant and anti-inflammatory properties.

Disruption of the balance between oxidant and antioxidant molecules is defined as oxidative stress. It is known that the oxidant–antioxidant balance may be disrupted by drugs used in cancer treatment, and that organ damage develops as a result of this. Oxidative stress is known to play a major role in pathophysiological processes, and various biomarkers are being evaluated to demonstrate and monitor this [53]. Three of these, and the most studied ones, MDA, GPx and SOD, were measured in this study in order to assess the effects of CPT-11 and NAC. In some studies, the effect of CPT-11 was investigated in various tissues, including intestinal mucosa, and it was observed that levels of MDA increased and that the activities of GPx and SOD decreased, in these tissues [53,54]. In this study, MDA levels increased, with suppression of GPX and SOD activities in rats treated with CPT-11. As a sulfhydryl donor group, it was determined that MDA levels were significantly reduced in animals that received NAC, which revealed its antioxidant property, and were at the same level as the animals in the control group. This suggests that NAC is effective in reducing MDA levels by scavenging lipid peroxyl radicals. Furthermore, we feel that the significant increase in GPx and SOD activities in the CPT-11 and NAC-applied group is also an important finding. Thus, it is necessary to elucidate the pathophysiological mechanism of the effect of CPT-11 on these enzyme levels.

## 5. Conclusions

In line with the findings obtained in this study, the protective effect of NAC can be attributed to the suppression of pro-inflammatory cytokine expressions caused by CPT-11, proteolytic enzyme activation, and the correction of the deteriorated oxidant–antioxidant balance. These findings were also supported by histopathological examinations. Toxicity in cancer treatment is the most important limiting factor in its treatment. Although many studies have been carried out to date, this problem still remains. Although CPT-11 has been used in many cancer treatments in recent years, its damage to hepatorenal and gastrointestinal tissues is significant. Therefore, we believe that these results encourage the design of further detailed cancer studies using NAC.

## Figures and Tables

**Table 1:** Changes in serum enzymes and metabolites in the sera of rats (mean ± SD).

	Control	NAC	CPT-11	CPT-11+NAC
Albumin (g/dL)	3.25 ± 0.25	3.27 ± 0.23	3.17 ± 0.25	2.91 ± 0.34
TP (g/dL)	6.10 ± 0.63	6.15 ± 1.82	5.78 ± 1.60	4.76 ± 0.40
ALP (U/L)	124.20 ± 54.96	137.2 ± 45.55	266.20 ± 57.47 ***^,††^	153.00 ± 43.78 ^§§^
ALT (U/L)	47.36 ± 11.27	74.58 ± 13.51	157.80 ± 29.16 ****^,††††^	85.91 ± 20.13 *^,§§§§^
AST (U/L)	60.69 ± 23.30	63.82 ± 20.94	140.80 ± 23.23 ****^,††††^	84.40 ± 19.59 ^§§^
LDH (U/L)	398 ± 140	727 ± 161	2392 ± 817 ****^,††††^	901 ± 236 ^§§§§^
BUN (mg/dL)	12.97 ± 2.32	14.87 ± 3.02	23.49 ± 3.68 ****^,†††^	17.86 ± 2.60 *^,†^
Creatinine (mg/dL)	0.47 ± 0.13	0.51 ± 0.15	1.06 ± 0.19 ****^, ††††^	0.56 ± 0.10 ^§§§§^
Amylase (U/L)	2096 ± 128	1990 ± 171	1910 ± 252	1909 ± 270
Lipase (U/L)	30.33 ± 2.25	27.33 ± 1.63	26.67 ± 1.86	27.33 ± 5.28

* *p* < 0.05 *** *p* < 0.001 **** *p* < 0.0001 compared with control; ^†^ *p* < 0.05 ^††^ *p* < 0.01 ^†††^ *p* < 0.001 ^††††^ *p* < 0.0001 compared with NAC; ^§§^ *p* < 0.01 ^§§§§^ *p* < 0.0001 compared with CPT-11.

**Table 2:** Changes in MMPs activities and TIMP-1 levels in the sera and tissues of rats (mean ± SD).

	Control	NAC	CPT-11	CPT-11+NAC
MMP-1 (Serum pg/mL; Tissues pg/mg Protein)				
Serum	2.31 ± 0.4475	1.895 ± 0.4842	7.053 ± 1.871 ****^,††††^	2.943 ± 1.06 ^§§§§^
Stomach	1.175 ± 0.5253	1.41 ± 0.4878	2.842 ± 0.975 ***^,††^	1.81 ± 0.3281 ^§^
Small Intestine	0.5817 ± 0.215	0.5533 ± 0.243	1.165 ± 0.2037 ***^,†††^	0.8033 ± 0.1646 ^§^
Liver	0.91 ± 0.3876	1.368 ± 0.2883	2.463 ± 0.5477 ****^,†††^	1.687 ± 0.3654 *^,§^
Kidney	1.572 ± 0.4782	1.551 ± 0.8503	3.873 ± 1.047 ***^,†††^	2.247 ± 0.5629 ^§§^
MMP-2 (Serum pg/mL; Tissues pg/mg Protein)				
Serum	21.51 ± 6.658	20.00 ± 4.484	53.63 ± 19.79 ***^,†††^	32.67 ± 4.73^§^
Stomach	1.393 ± 0.3101	1.645 ± 0.4054	2.552 ± 0.5098 ***^,††^	1.685 ± 0.4281 ^§§^
Small Intestine	1.06 ± 0.2866	0.945 ± 0.3526	2.423 ± 0.4816 ****^,††††^	1.615 ± 0.3868 ^†,§§^
Liver	0.905 ± 0.2818	0.9117 ± 0.3597	1.837 ± 0.3499 ^**,†^	1.188 ± 0.4857 ^§^
Kidney	0.8333 ± 0.528	1.103 ± 0.4106	2.265 ± 0.3682 ***^,††^	1.428 ± 0.4825 ^§^
MMP-8 (Serum pg/mL; Tissues pg/mg Protein)				
Serum	80.67 ± 12.90	84.75 ± 13.42	222.60 ± 68.85 ****^,††††^	114.00 ± 32.13 ^§§§^
Stomach	2.553 ± 1.557	3.007 ± 1.626	7.078 ± 2.104 ***^,††^	4.195 ± 0.9386 ^§^
Small Intestine	1.407 ± 0.5606	1.307 ± 0.3708	3.44 ± 0.7098 ****^,††††^	2.313 ± 0.4913 *^,†,§§^
Liver	3.542 ± 0.7431	4.722 ± 0.798	9.173 ± 1.768 ****^,†††^	6.305 ± 2.086 *^,§^
Kidney	4.475 ± 1.303	5.152 ± 0.8695	11.08 ± 1.861 ****^,††††^	6.343 ± 1.629 ^§§§§^
TIMP-1 (Serum pg/mL; Tissues pg/mg Protein)				
Serum	1210 ± 368.2	851.5 ± 221.8	322.3 ± 87.42 ****^,††^	742.7 ± 152.7 *^,§^
Stomach	1261.0 ± 320.8	1143 ± 241.1	685.9 ± 178.8 **^,†^	1088 ± 182.7 ^§^
Small Intestine	518.40 ±49.80	447.90 ± 73.52	109.70 ± 39.56 ****^,††††^	261.70 ± 73.67 ^****,†††,§§^
Liver	276.10 ± 80.72	274.90 ± 56.13	101.60 ± 24.29 ***^,†††^	190.20 ± 41.62 ^§^
Kidney	353.20 ± 81.03	307.40 ± 63.72	97.83 ± 21.72 ****^,††††^	190.10 ± 25.94 ***^,††,§^

* *p* < 0.05 ** *p* < 0.01 *** *p* < 0.001 **** *p* < 0.0001 compared with control; ^†^ *p* < 0.05 ^††^ *p* < 0.01 ^†††^ *p* < 0.001 ^††††^ *p* < 0.0001 compared with NAC; ^§^ *p* < 0.05 ^§§^ *p* < 0.01 ^§§§^ *p* < 0.001 ^§§§§^ *p* < 0.0001 compared with CPT-11.

**Table 3:** Scores of tissue homogenate (stomach, small intestine, liver, kidney) histomorphological measurements (Mean ± SD).

	Control	NAC	CPT-11	CPT-11+NAC
Stomach				
Degeneration of surface epithelium	2.31 ± 0.45	1.90 ± 0.48	7.05 ± 1.87 ****^,††††^	2.94 ± 1.06 ^§§§§^
Disruptions in gland structures	1.18 ± 0.53	1.41 ± 0.49	2.84 ± 0.98 ***^,††^	1.81 ± 0.33 ^§^
Congestion	0.58 ± 0.22	0.55 ± 0.24	1.17 ± 0.20 ***^,†††^	0.80 ± 0.16 ^§^
Small Intestine				
Villus degeneration	21.51 ± 6.66	20.00 ± 4.48	53.63 ± 19.79 ***^,†††^	32.67 ± 4.73 ^§^
Congestion in lamina propria	1.39 ± 0.31	1.65 ± 0.40	2.55 ± 0.51 ***^,††^	1.68 ± 0.42 ^§§^
Disruption of intestinal glands	1.06 ± 0.29	0.95 ± 0.35	2.42 ± 0.48 ****^,††††^	1.61 ± 0.39 ^†,§§^
Liver				
Degeneration in hepatocytes	80.67 ± 12.90	84.75 ± 13.42	222.60 ± 68.85 ****^,††††^	114.00 ± 32.13 ^§§§^
Degeneration in sinusoids	2.55 ± 1.56	3.01 ± 1.62	7.09 ± 2.10 ***^,††^	4.20 ± 0.94 ^§^
Kidney				
Degeneration in tubular cells	1210.0 ± 368.2	851.5 ± 221.8	322.3 ± 87.4 ****^,††^	742.7 ± 152.7 *^,§^
Congestion	1261.0 ± 320.8	1143.0 ± 241.0	685.9 ± 178.8 **^,†^	1088.0 ± 182.7 ^§^
Caste formation in tubules	518.4 ± 49.8	447.9 ± 73.5	109.7 ± 39.7 ****^,††††^	261.7 ± 73.7 ****^,†††,§§^

* *p* < 0.05 ** *p* < 0.01 ***p < 0.001 **** *p* < 0.001 compared with CTRL; ^†^ *p* < 0.05 ^††^ *p* < 0.01 ^†††^ *p* < 0.001 ^††††^ *p* < 0.0001 compared with NAC; ^§^ *p* < 0.05 ^§§^ *p* < 0.01 ^§§§^ *p* < 0.001 ^§§§§^ *p* < 0.0001 compared with CPT-11.

## Data Availability

The data that support the findings of this study are available from the corresponding authors upon reasonable request.
